# Telomere length, antioxidant status and incidence of ischaemic heart disease in type 2 diabetes

**DOI:** 10.1016/j.ijcard.2016.04.130

**Published:** 2016-08-01

**Authors:** Stefano Masi, Francesco D’Aiuto, Jackie Cooper, Klelia Salpea, Jeffrey W. Stephens, Steven J. Hurel, John E. Deanfield, Steve E. Humphries

**Affiliations:** a National Centre for Cardiovascular Prevention and Outcomes (NCCPO), Institute of Cardiovascular Science, University College London, UK; b Department of Clinical Gerontology, King's College London, UK; c Periodontology Department, Eastman Dental Institute, University College London, UK; d Division of Cardiovascular Genetics, British Heart Foundation Laboratories, Institute of Cardiovascular Science, University College London, UK; e Institute of Molecular Biology and Genetics, Biomedical Sciences Research Center “Alexander Fleming”, Athens, Greece; f Diabetes Research Group, College of Medicine, Swansea University, Swansea, UK; g Department of Endocrinology, University College London Hospital, London, UK

**Keywords:** Diabetes, Cardiovascular risk, Oxidative stress, Telomeres

## Abstract

**Background:**

Type 2 diabetes (T2D) is associated with an increased risk of ischaemic heart disease (IHD). An accelerated process of vascular ageing induced by an increased oxidative stress exposure is suggested as potential pathway accounting for this association. However, no studies have explored the relationship between markers of vascular ageing, measures of oxidative stress and risk of IHD in T2D.

**Objectives:**

To explore the association between plasma antioxidant status, marker of cellular ageing (leukocyte telomere length, LTL) and 10 years risk of IHD in patients with T2D.

**Methods:**

Between 2001 and 2002, 489 Caucasians subjects with T2D were enrolled at the diabetic clinic, University College London Hospital. Plasma total anti-oxidant status (TAOS) and LTL were measured by photometric microassay and RT-PCR, respectively. The incidence of IHD over 10 years was determined through linkage with the national clinical audit of acute coronary syndrome in UK.

**Results:**

At baseline, TAOS was associated with LTL (age adjusted: r = 0.106, p = 0.024). After 10 years, 61 patients developed IHD. Lower TAOS and shorter LTL at baseline predicted an increased IHD risk at follow-up (age adjusted: p = 0.033 and p = 0.040, respectively). These associations were independent of age, gender, cardiovascular risk factors, circulating levels of CRP and medication differences.

**Conclusions:**

Reduced TAOS and short LTL are interrelated pathways which predict risk of IHD in patients with T2D. Our findings suggest that antioxidant defences are important to maintain telomere integrity, potentially reducing the progression of vascular ageing in patients with T2D.

## 1 Introduction

Type 2 diabetes mellitus (T2D) is a chronic disease characterized by multiple metabolic derangements, which disrupt the balance between reactive oxygen species and antioxidant defences at the cellular level [1]. Antioxidant capacity of plasma is the primary measure and marker to evaluate the status and potential of oxidative stress in the body. Plasma contains many compounds, which function against the oxidative stressors in the body thus protecting the cell and cellular biomolecules from being damaged. The reduced antioxidant capacity described in patients with diabetes results in greater exposure to oxidative stress and subsequent damage to proteins, lipids, and DNA, which leads to a rapid deterioration of a broad range of cellular functions and premature cellular ageing [2], [3]. These mechanisms underpin the development of several diabetic complications, including ischaemic heart disease (IHD) [4]. T2D can therefore be regarded as a model of accelerated biological ageing due to increased levels of oxidative stress exposure, and the increased risk of IHD as a manifestation of premature vascular ageing [5].

Over the last ten years, epidemiological studies have suggested that peripheral blood leukocyte telomere length (LTL) can be a useful biomarker of cardiovascular ageing. Multiple reports [6], [7], [8], [9], including a recent meta-analysis and GWAS study [10], [11], suggested that LTL is on the causal pathways for IHD. The association between LTL and IHD is thought to be mediated by oxidative stress exposure which is currently considered to be an important driver of atherosclerosis and its complications [12] as well as to cause a faster LTL attrition [13]. However, the impact of a reduced antioxidant capacity on LTL and risk of IHD has not been explored in patients with T2D.

We have studied a well characterised cohort of patients with T2D in order to explore the relationship between a baseline measure of total serum antioxidant capacity and LTL with subsequent risk of IHD over 10 years.

## 2 Methods

### 2.1 Study sample

The University College Diabetes and Cardiovascular disease (UDAC) study comprises 1011 individuals, who were recruited consecutively from the diabetes clinic at UCL Hospitals in 2001–2. The study was designed to investigate the association between inflammatory/metabolic genes and biochemical risk factors implicated in IHD in patients with diabetes. The study has been described in detail elsewhere [14], [15]. All patients had type 1 or type 2 diabetes according to WHO criteria [16]. Anthropometric measures (height, weight and BMI), blood pressure and blood samples as well as information on smoking history and current medication use were collected during their routine diabetes clinic appointment. Our analysis focuses on the subgroup of individuals of individuals with a diagnosis of T2D, of Caucasian origin and with available measures of plasma total anti-oxidant status (TAOS), LTL and cardiovascular outcome (n = 489, Fig. 1S of Supplementary Material). The rationale for the restriction of the analysis to the T2D and Caucasian groups was to reduce the heterogeneity of our study sample, due to the known differences in the pathogenesis of cardiovascular complication between different types of diabetes [17] and the different LTL distributions and rates of attrition amongst ethnic groups [18], [19]. Further, despite multiple studies documented that LTL can predict the risk of IHD in White American and Caucasian populations, there are no reports as of yet on South Asian populations with or without diabetes. Ethical approval was granted by UCL/UCLH Ethics Committee and all subjects gave written informed consent.

### 2.2 Plasma total anti-oxidant status and cardiovascular risk factor assays

Plasma samples were collected within the 12-month recruitment period and stored immediately at − 80 °C. Plasma total anti-oxidant status (TAOS) was measured by Sampson's modification of Laight's photometric microassay [20], using 2.5 μL citrated plasma samples in 96-well ELISA plates. TAOS was selected as: a) it correlates with markers of oxidative damage in peripheral blood of patient with diabetes [20], [21]; b) there is already evidence supporting a different anti-oxidant status of patients with type 1 or type 2 diabetes when compared to healthy controls [22], [23]; c) it is associated with subclinical atherosclerosis coronary artery disease events in observational and longitudinal studies including patients with and without diabetes [21], [24]. Inter- and intra-assay coefficients of variation were 14.1% and 4.3%, respectively. Levels of total cholesterol, triglycerides, HDL cholesterol and HbA1c were assayed according to standard chemistry protocols [25]. LDL cholesterol was calculated by the Friedwald equation.

### 2.3 DNA extraction and LTL assay

Leukocyte DNA was extracted by the salting-out method [26]. Telomere length was measured using a validated quantitative PCR-based method as previously described [27]. Briefly, the relative telomere length was calculated as the ratio of telomere repeats to single-copy gene (SCG) copies (T/S ratio). For each sample the quantity of telomere repeats and the quantity of SCG copies were determined in comparison to a reference sample in a telomere and a SCG quantitative PCR, respectively. The raw data from each PCR was analysed using the comparative quantification analysis (Rotor-Gene 6000 software, Corbett Research Ltd., Cambridge, UK). All PCRs were performed on the Rotor-Gene 6000 (Corbett Research Ltd., Cambridge, UK). The coefficient of variation in repeated measurements was 5.6%.

### 2.4 Coronary heart disease data

Data on incident IHD disease was retrieved from the Myocardial Ischaemia National Audit Project (MINAP), held within the National Institute of Cardiovascular Outcome and Research (NICOR). This is a national registry of patients admitted to hospitals in England and Wales with acute coronary syndromes (ACS). It was established in 1998 to provide participating hospitals with a common mechanism for auditing performance against standards defined in the National Service Framework for Coronary Heart Disease [28]. Data collection began in October 2000 and by mid-2002 all acute hospitals in England and Wales were participating in the registry. The characteristics, organization, availability, data quality, validation and accessibility of cardiovascular outcome data contained in the MINAP have been previously described [29]. A new diagnosis of IHD disease was identified using hospital discharge records, markers of myocardial necrosis, results of coronary angiograms and coded electrocardiographic findings, in accordance with the internationally agreed definition of ST-segment elevation myocardial infarction (STEMI) [30] and acute coronary syndrome without persistent ST-segment elevation [30], [31], [32].

### 2.5 Statistical analysis

Mean values between groups were compared using two sample t-tests. Normality was tested using the Shapiro–Wilk test. Variables were log-transformed where necessary to normalise the distribution and geometric means and approximated standard deviations are reported for these variables with t-tests performed on the log-transformed data. Where the data could not be normalised, medians and interquartile ranges are presented and differences were tested using the Mann–Whitney U test. For categorical variables, chi-squared tests were used. Association between continuous variables was assessed by Spearman rank correlation. Adjustment was made for covariates by including them as terms in regression or logistic regression models. Particularly, a series of multivariable regression models were fitted to examine whether traditional CV risk factors and other potential confounders influenced the association observed between LTL and the risk of IHD disease. Results from three multiple regression models are reported: model 1 = age adjusted; model 2 = model 1 + adjustments for sex, HbA1c and smoking; model 3 = model 2 + adjustments for total cholesterol, blood pressure, C-reactive protein (CRP) and medications. Additionally, we explored whether further adjustment of model 2 for specific classes of anti-hypertensive medications (angiotensin converting enzyme, angiotensin receptor blockers or calcium channel blockers) had an impact on the association of TAOS or LTL with IHD. The α value for statistical significance for associations was set at 0.05. Analyses were performed with STATA version 13.

## 3 Results

### 3.1 Baseline characteristics

At baseline, the patients studied were overweight, exhibited suboptimal gluco-metabolic control, and relatively high levels of blood pressure (Table 1). The average TAOS was 44.8% [36.5–53.3] and it was higher in people with longer LTL (unadjusted: r = 0.093, p = 0.046; age adjusted: r = 0.106, p = 0.024) and higher levels of HDL-cholesterol, while it was reduced in patients with elevated glucose, HbA1c and triglycerides levels (Table 1). Furthermore, LTL was inversely associated with age (r = − 0.150; p = 0.002), while there were no differences based on gender or cigarette smoking distribution, nor was LTL associated with traditional cardiovascular risk factors including BMI, total cholesterol, HDL-cholesterol, systolic and diastolic blood pressure and HbA1c. Subjects with shorter LTL tended to have elevated levels of circulating CRP (Table 1).

### 3.2 Cardiovascular outcomes

**Fig. 1:**
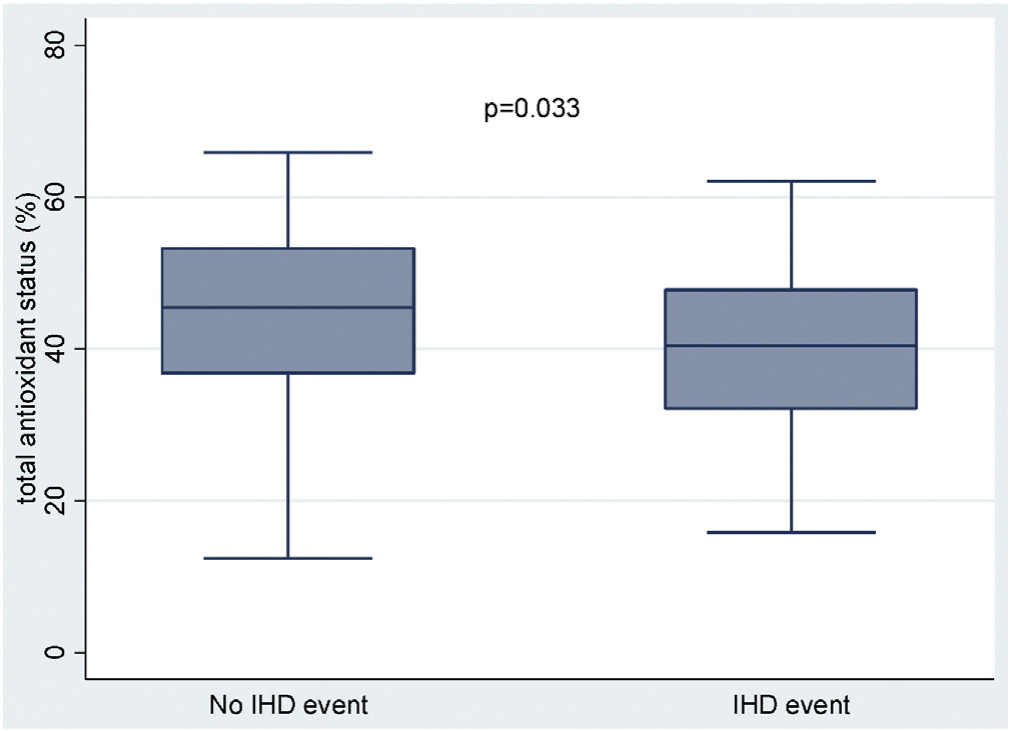
Box plot showing difference of TAOS at baseline between ischaemic and non-ischaemic groups (median and IQR); p = 0.033.

**Fig. 2:**
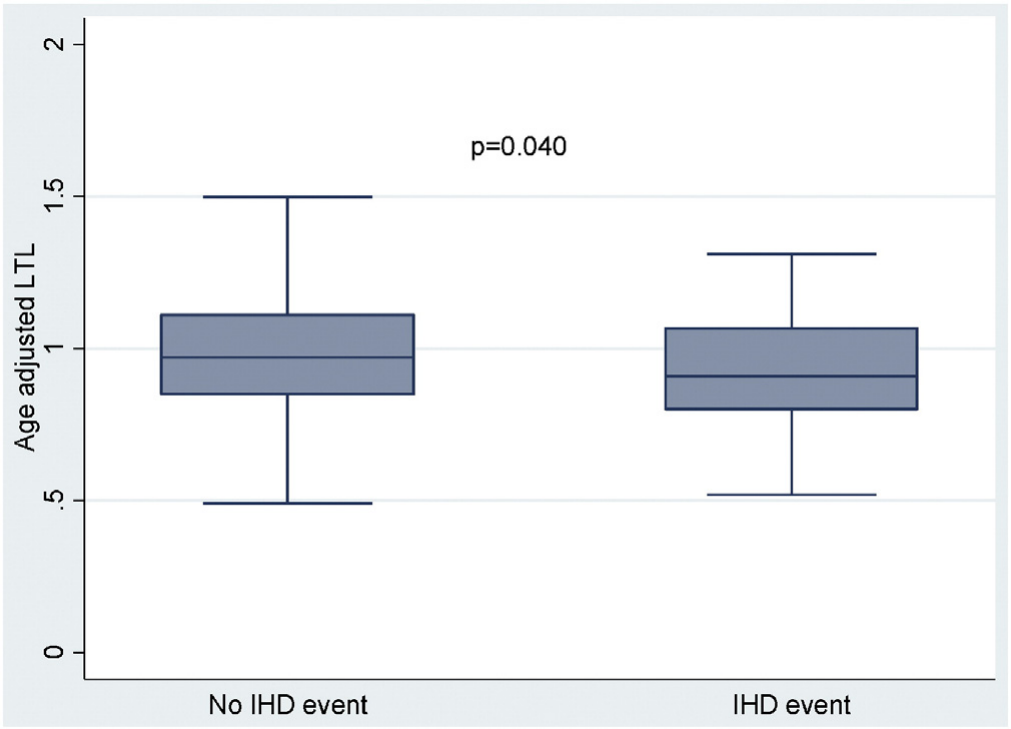
Box plot showing difference of LTL at baseline between ischaemic and non-ischaemic groups; analysis adjusted for age; p = 0.040.

After 10 years, 61 patients (12.5%) developed IHD disease. Patients with IHD had higher baseline BMI and CRP but lower levels of HDL-cholesterol compared to those in the non-ischaemic group (Table 2). Notably, the IHD disease group had lower baseline TAOS compared to the non-ischaemic group (unadjusted: p = 0.033; adjusted for age: p = 0.016) (Fig. 1). This association was not affected by adjustments included in model 2 (p = 0.028) and remained significant in the fully adjusted model (p = 0.022) (Table 3). Similarly, age-adjusted LTL was shorter in the IHD disease group compared to the non-ischaemic group (unadjusted: p = 0.040; adjusted for age: p = 0.039) (Fig. 2). This difference was not affected by adjustments included in model 2 (p = 0.034) and remained significant in the fully adjusted model (model 3, p = 0.020) (Table 4). Adjustment for medication use (Model 3 of Table 3, Table 4) as well as for different classes of anti-hypertensives did not materially affect the association between TAOS and IHD, nor the association between LTL and IHD (Tables 1S and 2S of Supplementary Material).

## 4 Discussion

This is the first study to explore the association between LTL, antioxidant capacity and subsequent risk of IHD disease in patients with T2D. We showed that baseline LTL was inversely related to TAOS and that shorter LTL and lower TAOS at baseline predicted IHD disease risk over 10 years, independently from traditional cardiovascular risk factors. This suggests that a reduced antioxidant capacity increases the risk of IHD in patients with T2D, potentially accelerating the vascular ageing process by damaging telomere sequences.

Previous reports have described associations between LTL and incidence of IHD in healthy populations [6], [7], [8]. In T2D, only observational studies have reported associations between LTL and prevalence of diabetes complications [33]. We now show that LTL can predict future incidence of IHD disease in prospective follow-up over 10 years. This is likely to be due to the unique ability of LTL to reflect an individual's cumulative exposure to inflammation and oxidative stress. Indeed, it is now well established that oxidative stress exposure increases LTL shortening and contributes to the initiation and progression of atherosclerosis. A higher oxidative stress exposure results in LDL oxidation, vascular inflammation and increased vulnerability of atherosclerotic plaques to rupture [12]. Similarly, oxidative stress exerts a major influence on telomere dynamics for two principal mechanisms. Firstly, the GGG triplets on the telomere sequence are highly sensitive to the hydroxyl radical [13]. Thus, conditions characterised by increased levels of oxidative stress exposure, such as T2D, can result in a longer stretch of telomeres being lost with each cell replication [13]. This has previously been confirmed by Sampson et al., who documented an association between oxidative DNA damage and monocyte telomere length in patients with T2D [34]. Secondly, in contrast to genomic DNA, telomeric DNA was reported to be deficient in the repair of single-strand breaks [35]. As a result, telomeres appear to be especially vulnerable to the accumulation of ROS-induced DNA-strand breaks [36].

We found an increased risk of IHD disease in T2D patients with reduced antioxidant capacity. A decreased antioxidant capacity is associated with an increase in oxidative stress which is thought to be on the causal pathway for diabetic vascular complications. Our study supports this hypothesis by demonstrating an inverse relationship of TAOS with risk of IHD disease risk. In line with our findings, Broedbaek et al. recently showed that higher urinary markers of nucleic acid oxidation are associated with increased mortality in newly diagnosed patients with T2D [37]. Despite this, the majority of clinical trials of antioxidants have failed to show significant improvement in CV outcomes in patients with diabetes [38], [39]. This may be due to the inability of exogenously provided compounds (like antioxidant vitamins) to reach intracellular compartments and prevent oxidative damage to key proteins, lipids and nucleic acids [40].

The association between TAOS and LTL with incident IHD disease was independent of traditional cardiovascular risk factors. For example, while people with lower TAOS had higher HbA1c and triglycerides with lower HDL-cholesterol, adjustment for these cardiovascular risk factors did not attenuate the association between TAOS and IHD. Similarly, higher CRP tended to be associated with shorter LTL, as expected [41], [42], [43], but addition of CRP to our fully adjusted model did not affect our results. This finding could be partially due to comparable cardiovascular risk factors burden between groups included in this study. Indeed LDL cholesterol levels were similar between the ischaemic and control groups, although use of statin was more prevalent in the former. This observation suggests that, whilst optimal treatment could normalize cardiovascular risk factors of people with T2D, this might not restore the antioxidant defences and counteract their impact on the cellular aging process. This hypothetical mechanism could explain the increased residual risk of cardiovascular events observed in people with T2D despite the improved cardiovascular risk factor burden.

Our study has limitations, which may lead to an underestimation of the strength of the associations between LTL and TAOS with incident IHD disease. Firstly, the primary outcome was IHD due to the limited information available on other atherosclerotic complications of diabetes. It is now well established that people with diabetes experience “silent” IHD during their lifetime. Secondly, the lack of data on non-cardiac causes of mortality precluded the opportunity to use an event-free survival approach in our statistical analysis. This, together with the similar follow-up length for all participants (range 9.3 to 10.5 years), led us to use logistic regression as preferred analytical models to explore the associations between TAOS and LTL with IHD. Thirdly, we could not perform measures of intracellular antioxidants. TAOS provides an estimation of total antioxidant capacity, which in turn is dependent on the contributions of albumin, bilirubin and urate. We cannot exclude therefore that measures of intracellular oxidative stress or the assessment of additional extracellular antioxidants could provide better estimation of the influence of antioxidant capacities on LTL and risk of future cardiovascular events. These factors do not attenuate, however, the importance of the biological associations emerging from our data. Larger epidemiological studies with multiple measures of LTL and oxidative stress will be necessary to provide a more accurate estimation of the associations between TAOS, LTL and IHD.

## 5 Conclusions

A single measure of antioxidant capacity and LTL predicted 10 years IHD risk in patients with diabetes. This association is likely to depend upon an increased damage of the telomere sequence in people with diabetes and suggests that a process of early vascular ageing induced by oxidative stress contributes to increase cardiovascular morbidity and mortality in diabetes.

## Author contribution

Design of original survey and participant recruitment: SEH, JWS, SJH; study design: SM, SEH, JED, FDA; telomere assay design and set up: SEH, KS; telomere assays: SM; biochemical assays: KS, JWS; statistical analysis: JK; data interpretation: SM, SEH, JED, FDA; manuscript preparation: SM, FDA; manuscript critical revision: SEH, JED, JK, KS, JWS, SJH.

## Conflict of interest

The authors report no relationships that could be construed as a conflict of interest.

## Figures and Tables

**Table 1:** Baseline characteristics of the study sample and their associations with TAOS and LTL.

Characteristics	N = 489	Association with TAOS		Association with LTL	
r	p	r	p
Age, years^e^	67 [24–91]	0.078	0.089	− 0.150	**0.002**
Smoking, %^c^	77 (16%)	− 0.0007	0.987	− 0.019	0.685
BMI, Kg/m2^b^	29.4 ± 5.6	− 0.058	0.204	− 0.0025	0.957
SBP^f^, mm Hg^b^	141 ± 19	0.075	0.104	0.061	0.192
DBP^f^, mm Hg^b^	79 ± 11	0.067	0.145	− 0.034	0.473
Total cholesterol, mmol/L^a^	5.15 ± 1.06	− 0.035	0.450	0.040	0.396
LDL, mmol/L^a^	2.79 ± 0.92	0.023	0.625	0.068	0.149
HDL, mmol/L^b^	1.29 ± 0.37	0.132	**0.004**	− 0.003	0.942
Triglyceride, mmol/L^b^	1.93 ± 1.09	− 0.178	**0.0001**	− 0.055	0.239
CRP, mg/L^b^	1.76 ± 1.51	0.074	0.106	− 0.092	0.051
Glucose, mmol/L^b^	10.00 ± 4.31	− 0.164	**0.0003**	− 0.054	0.251
Hba1c, % (mmol/mol)^b^	7.66 ± 1.64	− 0.100	**0.030**	0.011	0.817
TAOS, %^d^	44.8 [36.5–53.3]	–	–	0.106	**0.024**
Age adjusted LTL, T/S ratio^b^	0.97 ± 0.21	0.106	**0.024**	–	–
Statin treatment, %^c^	124 (26%)	− 0.010	0.833	0.022	0.646
BP lowering, %^c^	316 (65%)	− 0.0007	0.987	− 0.013	0.785

Apart from the association with age, all other associations with LTL were adjusted for age.

The α value for statistical significance for associations was set at 0.05.

a Mean ± standard deviation for normally distributed variables.

b Geometric mean ± approximate standard deviation for log-normally distributed variables.

c N (percentage) for binary variables.

d Median [interquartile range] for not normally distributed variables.

e Age is show as median [range].

f SBP: systolic blood pressure; DBP: diastolic blood pressure.

**Table 2:** Baseline differences between ischaemic and non-ischaemic groups.

Characteristics	Non-ischaemic N = 428	Ischaemic N = 61	p value
Age, years^e^	66 [24–91]	67 [44–84]	0.725
Smoking, %^c^	70 (17%)	7 (12%)	0.344
BMI, Kg/m2^b^	29·1 ± 5.6	30.9 ± 5.8	0.026
SBP^f^, mm Hg^b^	142 ± 18	140 ± 23	0.444
DBP^f^, mm Hg^b^	79 ± 11	76 ± 10	0.037
Total cholesterol, mmol/L^a^	5.18 ± 1.08	4.94 ± 0.97	0.094
LDL, mmol/L^a^	2.80 ± 0.93	2.69 ± 0.90	0.353
HDL, mmol/L^b^	1.30 ± 0.38	1.17 ± 0.29	0.004
Triglyceride, mmol/L^b^	1.90 ± 1.08	2.17 ± 1.08	0.087
CRP, mg/L^b^	1.70 ± 1.46	2.17 ± 1.90	0.041
Glucose, mmol/L^b^	9.94 ± 4.33	10.38 ± 4.15	0.471
Hba1c, % (mmol/mol)^b^	7.67 (60) ± 1.67	7.63 (60) ± 1.43	0.865
TAOS, %^d^	44.5 [36.9–53.3]	40.5 [32.3–47.8]	0.033
Age adjusted LTL, T/S ratio^b^	0.98 ± 0.21	0.92 ± 0.18	0.040
Statin treatment, %^c^	94 (22%)	30 (50%)	< 0.001
BP lowering, %^c^	268 (63%)	48 (79%)	0.019

Differences between ischaemic and non-ischaemic groups were assessed using unpaired t-test for normally or log-normally distributed variables. Where the data could not be normalised, medians and interquartile ranges are presented and differences were tested using the Mann–Whitney U test. χ [2] tests were used for categorical variables.

a Mean ± standard deviation for normally distributed variables.

b Geometric mean ± approximate standard deviation for log-normally distributed variables.

c N (percentage) for binary variables.

d Median [interquartile range] for not normally distributed variables.

e Age is show as median [range].

f SBP: systolic blood pressure; DBP: diastolic blood pressure.

**Table 3:** Multivariable models assessing differences of TAOS between non-ischaemic and ischaemic heart disease groups.

Models	Variables	Logistic regression	
OR^a^ (95% CI)	p values
Model 1	Age (1 year increase)	1.01 (0.98–1.03)	0.537
TAOS (1 quintile increase)	0.78 (0.64–0.95)	0.016
Model 2	Age (1 year increase)	1.01 (0.98–1.03)	0.555
Sex (female: male)	0.61 (0.34–1.11)	0.104
Hba1c (1 SD increase)	1.04 (0.78–1.38)	0.788
smoking (current vs. non)	0.67 (0.29–1.57)	0.361
TAOS (1 quintile increase)	0.80 (0.65–0.98)	0.028
Model 3	Age (1 year increase)	1.00 (0.97–1.03)	0.880
Sex (female: male)	0.45 (0.23–0.86)	0.017
Hba1c (1 SD increase)	0.98 (0.71–1.34)	0.984
Smoking (current vs. non)	0.73 (0.30–1.78)	0.494
SBP (1 SD increase)	1.13 (0.76–1.68)	0.552
DBP (1 SD increase)	0.64 (0.43–0.96)	0.030
Blood pressure medications	1.58 (0.79–3.19)	0.197
Lipid lowering medications	3.29 (1.77–6.11)	0.0002
CRP (1 SD increase)	1.48 (1.09–2.03)	0.013
TAOS (1 quintile increase)	0.78 (0.63–0.96)	0.022

a Odds ratio for a unit increase of the independent variable.

**Table 4:** Multivariable models assessing differences of LTL between non-ischaemic and ischaemic heart disease groups.

Models	Variables	Logistic regression	
OR^a^ (95% CI)	p values
Model 1	Age (1 year increase)	1.00 (0.98–1.03)	0.816
T/S ratio (1 SD increase)	0.74 (0.55–0.99)	0.039
Model 2	Age (1 year increase)	1.00 (0.98–1.03)	0.819
Sex (female: male)	0.56 (0.31–1.04)	0.067
Hba1c (1 SD increase)	1.02 (0.77–1.35)	0.880
Smoking (current vs. non)	0.59 (0.24–1.45)	0.248
T/S ratio (1 SD increase)	0.72 (0.53–0.97)	0.034
Model 3	Age (1 year increase)	0.99 (0.96–1.03)	0.705
Sex (female: male)	0.42 (0.21–0.83)	0.013
Hba1c (1 SD increase)	1.00 (0.72–1.39)	0.984
smoking (current vs. non)	0.72 (0.28–1.85)	0.497
SBP (1 SD increase)	1.09 (0.72–1.64)	0.681
DBP (1 SD increase)	0.67 (0.43–1.02)	0.062
Blood pressure medications	1.73 (0.82–3.62)	0.148
Lipid lowering medications	3.83 (2.02–7.25)	0.00004
CRP (1 SD increase)	1.29 (0.94–1.78)	0.121
T/S ratio (1 SD increase)	0.69 (0.50–0.94)	0.020

a Odds ratio for a unit increase of the independent variable.

## Appendix A Supplementary data

Supplementary material. Image 1
