# Supplementary material for: Telomere length, antioxidant status and incidence of ischaemic heart disease in type 2 diabetes
Source: Int J Cardiol. 2016 Aug 1;216:159–64. doi: 10.1016/j.ijcard.2016.04.130 (PMC4900130; doi:10.1016/j.ijcard.2016.04.130)
Supplement: Supplementary file 1 — Supplementary material. [file mmc1.docx]

**SUPPLEMENTARY DATA**

**Figure S1.** Cohort selection based on UDACS Study Flow Chart.

**
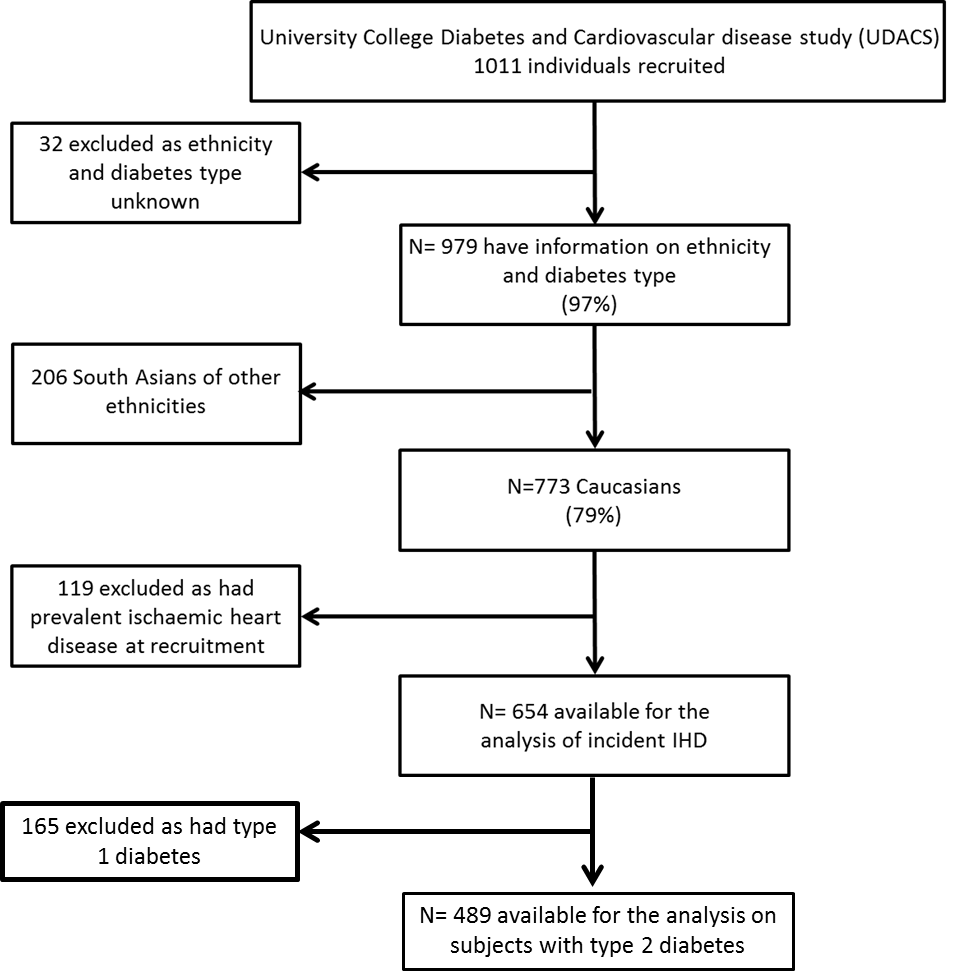
**

**Table S1.** Association between TAOS and IHD adjusted for medications (including SBP,DBP)

|  | Logistic regression | |
| --- | --- | --- |
| Adjusted for | OR* (95% CI) | P value |
| Model 2 | 0.80 (0.66-0.98) | 0.035 |
| Model 2 +ACEi | 0.80 (0.66-0.99) | 0.036 |
| Model 2 +ARBs | 0.79 (0.65-0.97) | 0.027 |
| Model 2 +CCBs | 0.80 (0.65-0.98) | 0.033 |

*Odds ratio for IHD for each quintile increase in TAOS

ACEi: angiotensin converting enzyme inhibitors; ARBs: angiotensin receptor blockers; CCBs: calcium channel blockers.

Model 2 = adjustments for age (Model 1) + sex, HbA1c and smoking + SBP + DBP

**Table S2.** Association between LTL and IHD adjusted for specific class of antihypertensive medications

|  | Mean telomere length | | Regression | Logistic regression | |
| --- | --- | --- | --- | --- | --- |
| Adjusted for | Non-ischaemic | Ischaemic | P value | OR* (95% CI) | P value |
| Variables in model 2 | 0.97 + 0.21 | 0.90 + 0.19 | P=0.025 | 0.69 (0.51-0.94) | 0.019 |
| Model 2 +ACEi | 0.97 + 0.21 | 0.90 + 0.19 | P=0.029 | 0.70 (0.51-0.95) | 0.023 |
| Model 2 +ARBs | 0.97 + 0.21 | 0.90 + 0.19 | P=0.019 | 0.67 (0.50-0.92) | 0.012 |
| Model 2 +CCBs | 0.97 + 0.21 | 0.91 + 0.19 | P=0.029 | 0.70 (0.51-0.95) | 0.021 |

*Odds ratio for IHD for each 1 SD increase in loge telomere

ACEi: angiotensin converting enzyme inhibitors; ARBs: angiotensin receptor blockers; CCBs: calcium channel blockers.

Model 2 = adjustments for age (Model 1) + sex, HbA1c and smoking + SBP + DBP
